# Case-based blended eLearning scenarios—adequate for competence development or more?

**DOI:** 10.1007/s40211-019-00322-z

**Published:** 2019-11-06

**Authors:** Patricia Pia Wadowski, Brigitte Litschauer, Tamara Seitz, Sebastian Ertl, Henriette Löffler-Stastka

**Affiliations:** 1 Department of Internal Medicine II, Division of Angiology, Medical University of Vienna, Vienna, Austria; 2 Department of Clinical Pharmacology, Medical University of Vienna, Vienna, Austria; 3 Department of Psychoanalysis and Psychotherapy, Medical University of Vienna, Währinger Gürtel 18–20, 1090 Vienna, Austria

**Keywords:** E‑learning, Curriculum, PTSD, Blended learning, Online Lernen, Lehrplan, PTBS, Präsenz-Seminare

## Abstract

**Background:**

Learning, competence development and scientific thinking in medicine need several strategies to facilitate new diagnostic and therapeutic ways. The optimal collaboration between creative thinking and biomedical informatics provides innovation for the individual patient and for a medical school or society. Utilizing the flexibilities of an e‑learning platform, a case based blended learning (CBBL) framework consisting of A) case based textbook material, B) online e‑CBL with question driven learning scenarios and C) simulated patient (SP) contact seminars was developed and implemented in multiple medical fields.

Real-life clinical cases were anonymized and transferred into an interactive and an interdisciplinary eLearning platform.

**Methods:**

As an example of the offered clinical teaching-case collection, an example of a psychiatric case for the disease “posttraumatic stress disorder (PTSD)” is presented: a 30-year-old man with a history of insomnia with difficulties in falling asleep and sleeping through, nightmares, nervousness and psychomotor restlessness. The students are challenged to identify possible differential diagnoses and further get to know the patient’s personal history (loss of relatives due to war, torture and flight from home country). Further, the students are guided through the principles of fear conditioning including translational aspects like neurotransmitter signaling of PTSD pathomechanism (translational and research aspects like dopamine transporter gene polymorphism, long term potentiation and synaptic signaling).

**Results/Conclusion:**

The case presentation comprises different learning aspects: First, declarative knowledge has to be acquired and collected in basic medical sciences, knowledge that is in fact available and can be accessed on the conscious and preconscious level in long-term memory. Second, associative learning leads to the formation of neuronal connections and is an important way of learning and discovering, founded in neural associations. Third, polythematic-crosslinking thinking is needed as ability to link information in a meaningful way. These steps are a typical intellectual ability of gifted learners and researchers that combine previously seemingly unrelated areas to each other and drive innovation.

## Zusammenfassung

**Grundlagen:**

Lern- und Forschungsprozesse sowie die Kompetenzentwicklung bei Studierenden brauchen in der Medizin verschiedene Strategien, um neue diagnostische und therapeutische Ansätze zu ermöglichen. Die optimale Vernetzung des kreativen Denkens und biomedizinischer Informatik ermöglicht Innovation einerseits für PatientInnen, andererseits für die medizinische Schule sowie die Gesellschaft. Auf der Basis der Flexibilität einer E‑learning Plattform wurde ein Fall-basiertes Lernprogramm, bestehend aus A) Fall-basiertem Lehrbuchmaterial, B) online – gestellten Fällen mit gezielten klinischen Fragen, und C) simuliertem PatientInnenkontakt im Seminar, implementiert. Klinische Fälle wurden anonymisiert und in eine interaktive und interdisziplinäre E‑Learning Plattform transferiert.

**Methodik:**

Als klinischer Lehrfall für die Erkrankung der posttraumatischen Belastungsstörung (PTSD) präsentieren wir einen 30-jährigen Mann mit Schlaflosigkeit, Ein- und Durchschlafstörungen, Albträumen, Nervosität und psychomotorischer Unruhe. Die Studierenden sind gefordert, mögliche Differentialdiagnosen anzugeben und erfahren die persönliche Geschichte des Patienten (Verlust der Angehörigen infolge von Krieg, Folter und Flucht aus dem Heimatland). Weiters werden die Studierenden durch die Grundlagen der Angstkonditionierung inklusive translationaler Aspekte wie Neurotransmitter-Signaling bei PTSD (translationale Forschungsaspekte wie Dopamin Transporter Gen Polymorphismus und Langzeitpotenzierung sowie Signalübermittlung an den Synapsen) geführt.

**Ergebnisse/Schlussfolgerung:**

Die Fallpräsentation umfasst verschiedene Lernaspekte: Als erstes muss deklaratives Wissen erworben werden und auf der Ebene der medizinischen Wissenschaft gesammelt werden, ein Wissen, dass jederzeit abrufbar und auf der bewussten und unbewussten Ebene im Langzeit-Gedächtnis zugänglich ist. Als zweites wird assoziatives Lernen trainiert, welches einen Beitrag zur Neuronenvernetzung leistet und einen wichtigen Grundstein für das Lernen und Entdecken, fundiert in neuronalen Verbindungen, darstellt. Als Drittes, ist das themenübergreifende Vernetzungsdenken notwendig, um Informationen zu verknüpfen. Diese Stufen zeichnen die typische intellektuelle Fähigkeit begabter Lerner und Forscher aus, welche zunächst scheinbar nicht zusammenhängende Gebiete verbinden und so auch Forschungsfragen vorantreiben.

## Case-based learning concept and implementation at the Medical University of Vienna

The integration of the interactive case-based e‑Learning into the Medical Curriculum of Vienna was initiated in 2014. Teachers and students provide real-life patient cases and background information, which is integrated into an online platform of the Medical University Vienna. Data are anonymized and transferred from the General Hospital Information Management into the established “Moodle”-E-learning database [1, 2]. Competence levels acquired by students are based on Dublin descriptors [3] and include the Austrian Catalogue of Competence levels for medical skills. Furthermore, compatibility is given with the CanMED framework, which sets definitions of the physicians’ roles (Medical Expert, Communicator, Collaborator, Manager, Health Advocate, Scholar and Professional) and thematic groups of competencies [4].

Over 100 different cases in many different specialties like internal medicine, trauma surgery, orthopedics, dermatology, pediatric care, laboratory medicine, microbiology, and psychiatry are offered. The level of complexity and learning objectives are also differing, ranging from diagnosing ordinary infections to treating and managing very complex cases interdisciplinarily within an biopsychosocial framework.

As an example, we provide a clinical psychiatric case of posttraumatic stress disorder (PTSD), a 30-year-old refugee suffering from typical disease-related symptoms, to illustrate the design of our cases. On the basis of the Bloom’s taxonomy criteria [5], the learning scenarios are constructed as follows:

1. *Knowledge:* The patient presents with a history of insomnia with difficulties in falling asleep and sleeping through, nightmares, nervousness and psychomotor restlessness. In the next step, the student is challenged to identify possible differential diagnoses and further gets to know the patient’s personal history (loss of relatives due to war, torture and flight from home country). In the e‑case-presentation links e.g., to guidelines for history taking, or epidemiological studies are given to provide material for further reading, training or repetition.
2. *Comprehension: *Through presentation of visual graphs and guided through a question-based feedback-system the student learns to understand disease-related pathomechanisms: The classical conditioning model is recapitulated with the presentation of the conditioned stimulus to an animal, secondary coupling with the unconditioned stimulus and fear as the result of sole presentation of the previously neutral conditioned stimulus [6]. In this context, the amygdala as central site of plasticity and fear learning is presented [7]. Furthermore, pathways involved in fear conditioning like signaling through the inhibitory GABAergic and stimulatory glutamatergic connections [8, 9], or dopaminergic and glucocorticoid pathways are discussed [10, 11]. Herewith, a model representing molecular basic principles, precisely depicted through visual data and connected with a literature database created for the case, is generated and serves as a central translational element of this case-based exercise. In this section of the e‑case many interactive questions are inserted always providing the arguments why something is right or wrong. The students can utilize this section interatively, links to reading material or lectures on basic knowledge are provided in order to be able to attend the specific lecture and ask the experts directly.
3. *Application: *Based on the previous information, the student has to identify the trauma type of the patient according to Leonie Terr [12]. In addition, students are tested about predictors of the development of PTSD [13].
  Clinical decision-making is required from the student at the end of the exercise to initiate adequate therapy and to decide for the best integrative options, whereby interdisciplinary therapy is essential. Cases are further presented in seminars where students are supervised and have the opportunity to train communicative skills. The topic of exploration is then trained in supervised seminars, further (psycho)therapeutic consequences are focused in supervised small group seminars with simulated patients. The main advantage of training within an affectively wholesome and secure learning environment where students can work, try, experience all challenging demands of working with severely disturbed patients, is that they also get feedback on their performance especially from the patient’s view given by a sufficiently trained simulated patient.
4. *Synthesis: *To study disease complexity and associated morbidity, the case points to incipient risk factors like hypertension. It is supposed that repetitive or continuous stress disturbs the autonomic nervous system, the hypothalamic–pituitary–adrenal axis, metabolism and further the cardiovascular and immune systems [14, 15]. There is a high relationship of PTSD and cardiovascular disease, psychosocial factors being even a higher risk factor than for e.g. hypertension, diabetes or obesity [16]. However, PTSD and depression is strongly associated with cardiovascular risk factors [17, 18]. Herein, endothelial dysfunction is proposed to be one of the underlying causes [15]. A pro-inflammatory state due to lower peripheral cortisol levels [19] is discussed leading to endothelial activation [15]. Furthermore, a decrease in nitric oxide [20] and increase in oxidative stress [21] as well as vasoconstrictors [22, 23] are proposed as contributing factors [15]. Through the description of the complex disease pattern, cross-linking thinking is trained, and current research questions are pointed out. Each student has to conduct his or her own portfolio and work out several questions, own thoughts and research ideas, sometimes research gaps.
5. *Analysis: *PTSD and associated co-morbidities remain a major burden and preventive measures are of great importance. Hence it is necessary for physicians to be alert to signs of cardiovascular risk factors and to initiate adequate medical treatment. It could be shown that residents included in case-based learning programs showed higher baseline knowledge in their field [24]. The e‑learning cases are further valuable in consistent representation of best practice cases linking to updated guidelines and consensus statements. A further step integrated in the Viennese curriculum is the gathering of clinical practice in seminars with actors simulating key clinical symptoms and lectures for each topic represented in the online case collection [25]. “Gathering of clinical experience” via bed-side training and with simulation situations, training of clinical reasoning, functions as a best-practice example, that can be provided via eLearning in a consistent way. By providing feedback, metacognition is trained as a process to avoid cognitive errors and promote analytic reasoning [26]. Reflexion on feedback from teachers, colleagues, researchers has to be written into the portfolio.
6. *Evaluation: *The programmed question-driven feedback-system serves at each step of the case as checkpoint to allow critical reflection and developing of metacognitive competences. Recapitulation of previously presented information should serve as a basis for plasticity in synaptic connections and hippocampal long-term potentiation [27].

Following that, interactive questions are constructed to train and test learning objectives as well as create procedural knowledge. The interactive questions focus on network or associative knowledge with previously learned textbook knowledge while training clinical reasoning by applying the learned contents. The questions focus on 1) recognizing symptom patterns and red flags, 2) clinical reasoning—understanding modes of operation for creating and arguing diagnostic hypotheses, and 3) clinical decision making—making diagnostic and therapeutic conclusions. At the end of a year or of a certain curriculum element the students have to pass an Objective Structured Clinical Examination (OSCE) to show their performance and competence. Translational knowledge is evaluated in the portfolios.

Linguistically and for argumentation strategies, an important dimension is defined in requiring students to answer why which clinical hypotheses are in/correct, why they would proceed with a therapy. Additionally, field specific foci with recent research impulses are set by educators in the field. Examples of these foci are dimensions of communication in psychiatry, interdisciplinary referral communication in medicine, and the systematic approach to a clinical examination for e.g., traumatology.

The aim of the case-based eLearning is to transform declarative knowledge into procedural knowledge, so that students are already accustomed to procedural algorithms for the patient encounter [2, 26].

## Beyond teaching: digital interface for clinics, teaching and research

International studies and curricular reforms emphasizing new teaching methods [26] described case-based learning to be the best way for preparation in clinical problem solving and to be an effective lecture format, in order to gain improvements in declarative as well as procedural knowledge via fostering the understanding of (patho)physiological concepts. A strengthened understanding of pathoplastic concepts could be developed and an improvement of grades among students could be shown (e.g., in the US in the USMLE-scoring) [24, 28, 29]. The USMLE, as a test for medical licensure in the US, is a tool to measure the ability to apply knowledge, concepts and principles. Skills, that are essential for a safe and effective patient care [30]. This 3 step test ensures, that all doctors in the US who have passed, shall have the same competence level.

In addition, controlled E‑learning cases promote students’ satisfaction [31] and are suggested as an instrument for burnout prevention in medical students [32].

At the Medical University of Vienna, the preparation and discussion of E‑learning cases has led to a networking platform of students, postgraduates, clinicians and teachers. The working groups discuss clinical and translational aspects in order to foster research cooperation. The online e‑learning platform serves as basis for a database of cases constructive for postgraduate teaching and interdisciplinary research questions.

Continuous evaluation, technical improvements as well as updates on current clinical guidelines are performed to set high standards, foster effective information transfer and ensure persistent student satisfaction [33]. The latter was the advance of business schools, which have a higher emphasis on creative problem-solving, career counseling and training in resilience [32].

The e‑collection of academic cases with a step-wise feedback system provides a continuously actualized platform to link newest insights of basic research to clinical practice thus familiarizing students with research questions and the current research approach.

It must be highlighted, that the advantage of a case-based learning approach is underrated [34] and due to missing research in the field of students’ attitudes, knowledge levels and competencies, an ongoing student tracking project is currently established and in progress.

### Take home message

Recent research aspects were integrated in the case presentations to enable translational thinking. Satisfaction with this kind of learning led to formation of innovative learning-platforms and publication groups that began to develop critical reflection on curricular development, patient-centered clinical reasoning processes and research questions—both in students and teachers.

It has to be highlighted, that further research in the field of CBL is necessary.
